# Assessing the risks of short-term exposure to ambient air pollutants on COVID-19 hospitalizations in Tehran, Iran: a time-stratified case-crossover approach

**DOI:** 10.3389/fpubh.2025.1514721

**Published:** 2025-06-03

**Authors:** Mojtaba Sepandi, Yousef Alimohamadi, Mohammad Sakhaei, Amir Mirshafiee, Kolsoom Alimohamadi

**Affiliations:** Health Research Center, Life Style Institute, Baqiyatallah University of Medical Sciences, Tehran, Iran

**Keywords:** COVID-19, air pollutants, hospitalization, relative risk, case-crossover, DLNM model

## Abstract

**Purpose:**

This study aimed to evaluate the impact of both cumulative and non-cumulative exposure to air pollutants on hospitalizations due to Coronavirus Disease 2019 (COVID-19) in Tehran.

**Methods:**

A time-stratified case-crossover approach was employed to estimate the relative risks and assess the attributable fraction and attributable number of COVID-19 hospitalizations associated with air pollution exposure. Data on hospitalizations were collected from a teaching hospital in Tehran between March 20, 2020, and September 20, 2022, and were categorized by gender and age. Air pollution data including fine particulate matter (particles with a diameter less than 2.5 micrometers), nitrogen dioxide, sulfur dioxide, coarse particulate matter (particles with a diameter less than 10 micrometers), ozone, and carbon monoxide were obtained from the Environmental Protection and Air Quality Control Organization of Tehran. Quasi-Poisson conditional regression and distributed lag non-linear models were applied to estimate the relative risk of hospitalizations associated with pollutant exposure.

**Results:**

The findings indicate a significant association between exposure to fine particulate matter, nitrogen dioxide, and ozone with increased COVID-19 hospitalizations. The estimated relative risks for hospitalizations were 1.36 (95% confidence interval: 1.15–1.62), 1.17 (95% confidence interval: 1.07–1.29), and 1.37 (95% confidence interval, 1.19–1.58), respectively. No significant association was observed between coarse particulate matter exposure and hospitalizations. The number of hospitalizations attributed to ozone (6,000 cases) and nitrogen dioxide (3,300 cases) exceeded those associated with other pollutants.

**Conclusion:**

This study highlights the impact of air pollution on increased hospitalization risk for COVID-19. These findings underscore the urgent need for health authorities to implement stringent air quality regulations and pollution control measures to mitigate the adverse health effects of air pollution.

## 1 Introduction

Air pollution is widely recognized as a major global environmental health risk, contributing significantly to increased healthcare expenditures and premature mortality (1). According to the World Health Organization (WHO), ambient (outdoor) air pollution was responsible for approximately 4.2 million premature deaths worldwide in 2019, primarily due to ischemic heart disease, stroke, chronic obstructive pulmonary disease (COPD), lung cancer, and acute respiratory infections (2, 3).

The respiratory system is particularly vulnerable to the harmful effects of air pollutants. Numerous studies have established a strong association between exposure to elevated concentrations of ambient pollutants and the exacerbation of respiratory diseases (4–7). For instance, research has linked pollutant exposure to chronic bronchitis and a higher prevalence of respiratory conditions in children (8). WHO estimates that air pollution contributes to 29% of lung cancer deaths and 43% of deaths related to COPD (9).

Global health has suffered dramatically in the last few years due to COVID-19, a respiratory illness (10). As of early 2024, over 770 million confirmed cases and nearly 7 million deaths have been reported worldwide. Air pollution has been proposed as a contributing factor in the transmission, severity, and mortality associated with COVID-19. Several studies suggest that SARS-CoV-2 particles can remain suspended in aerosols, potentially facilitating airborne transmission under certain environmental conditions (11–14).

Moreover, recent cohort and ecological studies have demonstrated significant associations between exposure to ambient air pollution particularly fine particulate matter (PM₂.₅) and nitrogen dioxide (NO₂) and an increased risk of COVID-19-related hospitalization and mortality (15–17). For example, a U. S.-based cohort study reported that PM₂.₅ exposure during the 7 days prior to a COVID-19 diagnosis was associated with a 1.18-fold increased risk of hospitalization (95% CI: 1.17–1.19) (15). Similar patterns have been observed in various global contexts, including OECD countries and during waves involving variants of concern, such as the Delta variant (18, 19).

Despite the growing body of international evidence, there remains a scarcity of research investigating this association in Iran particularly in Tehran, a megacity of more than 8.5 million residents and one of the most polluted urban centers globally. According to WHO estimates, air pollution adversely affects the health of nearly 1 million people annually in Tehran. In this context, rigorous epidemiological research is essential to assess the health impacts of air pollution in this high-risk setting.

Unlike many prior studies that primarily employed time-series models, the present study utilizes a time-stratified case-crossover design to examine the short-term effects of air pollutants on COVID-19 hospitalizations. This design allows for effective control of long-term trends and seasonal variations, thereby minimizing confounding (20). In addition, we estimated attributable fractions and the number of hospitalizations attributable to each pollutant, providing practical, policy-relevant metrics to assist health authorities in identifying and prioritizing environmental risk factors both during and beyond the COVID-19 pandemic. Therefore, the aim of this study is to quantify the short-term association between air pollution exposure and COVID-19 hospitalizations in Tehran, and to estimate the health burden attributable to specific pollutants in order to inform targeted public health interventions.

## 2 Methods

The present study employed a time-stratified case-crossover design in Tehran to estimate both cumulative and non-cumulative relative risks (RRs) of COVID-19 hospitalizations (CHs) associated with exposure to air pollutants across various lag periods. In addition, using this modeling framework, we calculated key epidemiological indicators, including the attributable number (AN) and attributable fraction (AF) of CHs linked to air pollution exposure. Detailed descriptions of the model specification and data collection procedures are provided in the following sections.

### 2.1 Data collection

We collected daily counts of COVID-19 hospitalizations from Baqiyatallah Teaching Hospital in Tehran over the study period, spanning from March 20, 2020, to September 20, 2022. Hospitalization data were stratified by age and sex, with age categorized into two groups: individuals younger than 65 and those aged 65 and above.

Daily concentrations of six major air pollutants including fine particulate matter (PM₂.₅), nitrogen dioxide (NO₂), sulfur dioxide (SO₂), coarse particulate matter (PM₁₀), ozone (O₃), and carbon monoxide (CO) were obtained from the Environmental Protection Organization^1^ and the Air Quality Control Company.^2^ While air pollution data were originally recorded hourly at monitoring stations, we aggregated them into different averaging periods based on standard guidelines: 24-h averages for PM₂.₅ and PM₁₀, 8-h moving averages for O₃ and CO, and maximum 1-h averages for NO₂ and SO₂. These averaging approaches follow recommendations by the World Health Organization (WHO) and the U.S. Environmental Protection Agency (EPA), reflecting the distinct atmospheric behavior, health effects, and emission sources of each pollutant.

Only monitoring stations with at least 75% valid daily data were included in the analysis. Additionally, outliers and negative values were excluded. Meteorological variables used as confounding factors were retrieved from the Iran Meteorological Organization and measured by three monitoring stations in Tehran. Given the absence of strict nationwide lockdown measures during the study period in Iran, we assumed relatively consistent levels of population mobility and pollutant exposure throughout the entire duration. Therefore, no specific adjustments were made for potential lockdown effects.

There were no missing data for hospitalizations or meteorological parameters. The air pollution dataset had less than 10% missing values, which were handled using linear interpolation, a standard method in environmental epidemiology. This approach ensures the temporal continuity of the dataset while minimizing potential biases.

### 2.2 Statistical analysis

We employed a conditional quasi-Poisson regression model integrated with distributed lag non-linear models (DLNM) to assess the short-term associations between air pollutant exposure and CHs (21). To minimize potential overlap bias in the estimates, we implemented a fixed, non-overlapping time-stratified design (22, 23). In this approach, the day of hospitalization, along with the same weekdays in the weeks before and after the event within the same calendar month, was selected as the control period. Unlike the conditional logistic model, the quasi-Poisson approach conditions stratum-specific parameters within the model rather than estimating them directly.

The conditional quasi-Poisson model offers simplicity in implementation and accounts for both overdispersion and autocorrelation in the outcome variable (24). DLNM, on the other hand, enables modeling of nonlinear and lagged effects of exposure, capturing delayed associations across time. The final model equation is expressed as:


(1) $$\begin{array}{l} \log(Y_{t})=\alpha +\mathrm{Cb}(\text{Pollutan}t_{t,l})+\mathrm{NS}(\text{Temperature},\mathrm{Df}=3)\\ +\mathrm{NS}(\text{Humidity},\mathrm{Df}=3)+\mathrm{NS}(\text{Wind Speed},\mathrm{Df}=3)\\ +\text{Holiday}+\varepsilon_{t}\; \end{array}$$

Here, *Y_t_*​ is the number of CHs on day *t*, *α* is the intercept, and *Cb* denotes the cross-basis function capturing both the exposure-response and lag-response dimensions (21) (Equation 1). Natural spline (*NS*) functions were applied to temperature, humidity, and wind speed to control for potential nonlinear confounding effects, with three degrees of freedom for each. The binary “Holiday” variable accounts for national holidays, and **ε**_**t**_ is the error term (25). The cross-basis function allows for defining linear or nonlinear relationships independently for the exposure and lag dimensions (23, 26, 27). In this study, we utilized natural cubic *B*-splines for both dimensions, as supported by previous research and sensitivity analyses (28, 29).

To estimate both cumulative and non-cumulative relative risks, we applied a pollutant-specific increment approach using reference values derived from percentiles of the pollutant distribution. The following increments were used: 10 μg/m^3^ for PM₂.₅ and PM₁₀, 10 ppb for O₃ and NO₂, 1 ppb for SO₂, and 1 ppm for CO (30). The reference values were set at the 25th percentile for PM₂.₅, CO, and O₃, and the 50th percentile for PM₁₀, NO₂, and SO₂.

For the exposure-response function, we placed three knots at the 10th, 75th, and 90th percentiles. For the lag-response function, we used two knots equally spaced on a logarithmic scale (31). The number and placement of knots, as well as the degrees of freedom, were selected based on sensitivity analyses using the Akaike Information Criterion (AIC).

The maximum lag period used to estimate the relative risks was 14 days. This period approximately corresponds to the typical incubation time and the interval between symptom onset and hospitalization for COVID-19. Since COVID-19 symptoms often appear within 2 to 14 days following exposure, and hospitalization typically occurs within this timeframe for severe cases, a 14-day lag is clinically justifiable. Moreover, the majority of previous epidemiological studies have adopted this lag period to assess short-term exposure effects. Therefore, to maintain consistency with prior research and support future meta-analyses, we selected a 14-day lag in our study. Additionally, sensitivity analyses were conducted for longer periods, including up to 21 days, to test the robustness of the results.

#### 2.2.1 Attributed risk (fraction/number)

In epidemiological research, the attributable fraction (AF) quantifies the proportion of disease cases that can be attributed to exposure to a specific risk factor, under the assumption of a causal relationship. It reflects the potential reduction in disease burden that could theoretically be achieved if the exposure were eliminated. Within the distributed lag non-linear model (DLNM) framework, we estimated both the AF and the attributable number (AN) of COVID-19 hospitalizations associated with air pollution exposure over the study period. This approach accounts for both the delayed and non-linear effects of air pollutants, providing a more accurate estimate of their contribution to the total disease burden. These metrics are particularly valuable for public health planning, as they help quantify the impact of environmental exposures and support the prioritization of mitigation strategies (32, 33). These metrics were computed for the overall range of pollutant concentrations, as well as within specific exposure categories: low (10th–25th percentile), medium (25th–50th percentile), high (50th–90th percentile), and very high (90th–99th percentile).

AF was calculated using the following equation:


(2) $$\;\mathrm{AF}=1-\exp(-\sum_{\ell =\ell 0}^{L}\beta_{x_{t-\ell },\ell })\;$$

Where AF is the attributable fraction (23) of COVID-19 hospitalizations related to x exposure at time t, βx represents the risk associated with exposure (x) at time t corresponding to the relative risk in this research (32). Here, we employ a backward perspective that calculates the attributable risk over past lags (t − ℓ0,…,t − L) (Equation 2). The upper and lower limits of the confidence interval were calculated using the Monte Carlo method (34).

The AN of COVID-19 hospitalizations to air pollutants can be obtained based on the AF and the COVID-19 hospitalizations in time t (days). The general formula of *AN* is as follows:


(3) $$\;AN_{x,t}=AF_{x,t}*n_{t}\;$$

In Equation 3, *AN* is the number of hospitalizations attributable to pollutant x on day t, and n_t_ is the observed count of COVID-19 hospitalizations on that day. Because AF and AN are expressed as proportions and counts, respectively, they provide interpretable indicators for public health planning and policy-making particularly in identifying priority pollutants for intervention during respiratory epidemics such as COVID-19. All statistical analyses were conducted using R software (version 4.2.2), with statistical significance set at *p* < 0.05.

## 3 Results

### 3.1 Descriptive analysis

A total of 21,711 CHs were recorded during the study period. Among these, 12,251 (56%) were male and 9,460 (44%) were female. In terms of age distribution, 27% of patients were aged 65 years and older, while 73% were under the age of 65. Table 1 presents a descriptive analysis of the study variables, including the mean, minimum, and maximum values for CHs and independent variables such as air pollutants and meteorological parameters.

**Table 1:** Descriptive statistics of COVID-19 hospitalizations and daily average values of the studied variables in Tehran, Iran, during 2020–2022.

Variables		Mean	SD	Min	Max	P25	P50	P75	P90
COVID-19 hospitalization (CHs)	Total	23.73	21.87	0.00	104.00	7.00	17.00	34.50	55.00
	Male	13.39	12.49	0.00	61.00	3.00	9.00	21.00	32.00
	Female	10.34	9.91	0.00	49.00	3.00	8.00	15.00	25.00
	≥65	6.40	5.06	0.00	25.00	2.00	6.00	10.00	13.00
	<65	17.33	17.93	0.00	88.00	4.00	11.00	25.00	42.00
Pollutants	O_3_ (ppb)a	43.03	20.29	5.31	98.34	27.53	43.54	56.33	69.84
	CO (ppm)b	3.03	0.48	1.86	4.90	2.67	2.99	3.34	3.67
	NO_2_ (ppb)	75.54	18.29	31.67	154.51	61.83	73.81	86.73	100.53
	SO_2_ (ppb)	15.28	8.96	4.07	78.79	9.96	12.81	17.55	24.25
	PM_10_ (μg/m^3^)c	81.03	42.94	14.11	598.14	58.08	74.59	92.40	120.32
	PM _2.5_ (μg/m^3^)	30.68	14.97	6.49	142.56	21.98	26.81	34.82	50.06
Weather factors	Temperature (°c)	19.58	9.52	−1.30	34.63	10.78	20.93	28.51	30.91
	Humidity (%)	33.37	18.31	9.02	96.96	19.61	28.62	43.25	60.66
	Wind speed (m/s)	2.26	0.94	0.51	6.13	1.68	2.11	2.63	3.47

^a^parts per billion, ^b^parts per million, ^c^micrograms per cubic meter.

The maximum number of daily hospitalizations observed was 104, with a mean of 23.73 cases per day (SD = 21.87). The mean concentrations of PM₂.₅, NO₂, SO₂, PM₁₀, O₃, and CO were 30.68 μg/m^3^, 75.54 ppb, 15.28 ppb, 81.03 μg/m^3^, 43.03 ppb, and 3 ppm, respectively. According to the guidelines set by the World Health Organization (WHO) and the U. S. Environmental Protection Agency (EPA), the recommended limits for these pollutants are as follows: 15 μg/m^3^ (WHO) and 35 μg/m^3^ (EPA, 24-h) for PM₂.₅; 25 μg/m^3^ (WHO) and 100 ppb (EPA, 1-h) for NO₂; 40 μg/m^3^ (WHO) and 75 ppb (EPA, 1-h) for SO₂; 45 μg/m^3^ (WHO) and 150 μg/m^3^ (EPA, 24-h) for PM₁₀; 100 μg/m^3^ (WHO) and 70 ppb (EPA, 8-h) for O₃; and 9 ppm (EPA, 8-h) for CO.

Based on these standards, the daily average concentrations of PM₂.₅, PM₁₀, and NO₂ exceeded the WHO-recommended thresholds. The average daily temperature, wind speed, and relative humidity during the study period were 19.58°C, 2.26 m/s, and 33.37%, respectively.

### 3.2 Time trend

During the investigation period, the number of CHs and air pollutant levels exhibited noticeable temporal patterns. CHs initially declined in the spring of 2020 but sharply increased by the summer of 2021, with the highest number recorded in 2021. This trend aligns with national reports showing elevated hospital emergency triage activity in Iran since the onset of the SARS-CoV-2 pandemic (March 2019). Most pollutants, including PM₂.₅, PM₁₀, NO₂, SO₂, and CO, followed similar trends over time. In contrast, O₃ displayed a different temporal pattern. These variations are illustrated in Supplementary Figure 1.

### 3.3 Total CHs

**Figure 1:**
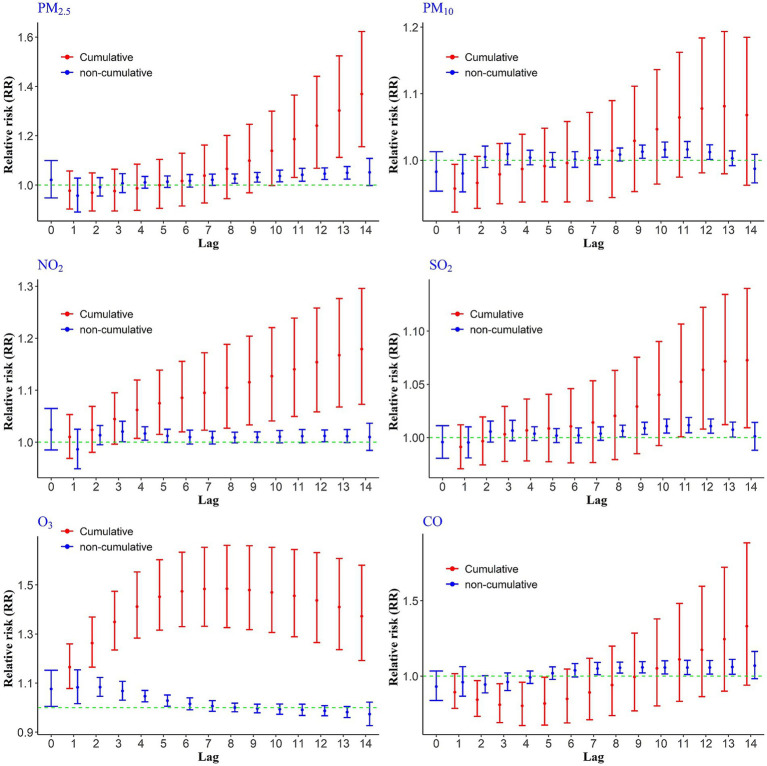
Cumulative and non-cumulative relative risks of CHs due to air pollutants across different lag during 2020–2022.

To evaluate the short-term effects of air pollutants on CHs, we analyzed both cumulative and non-cumulative relative risks across various lag periods. The results, illustrated in Figure 1, demonstrate that PM₂.₅ exhibited the highest non-cumulative RRs between lags 8 to 14, reaching a peak at lag 14 with an RR of 1.05 (95% CI: 0.99, 1.10) per 10 μg/m^3^ increase in concentration. For cumulative exposure, the most pronounced effect of PM₂.₅ occurred between cumulative lags 11 to 14, with the highest RR observed at lag 14 (RR = 1.36; 95% CI: 1.15, 1.62).

Other pollutants also showed statistically significant associations at certain time lags. For instance, a 10 ppb increase in NO₂ was associated with elevated non-cumulative RRs at lags 3, 4, and 12, with the highest value at lag 3 (RR = 1.02; 95% CI: 1.00, 1.04). In terms of cumulative exposure, NO₂ showed a consistent rise in relative risk from lag 4 through lag 14, with the maximum cumulative RR observed at lag 14 (RR = 1.17; 95% CI: 1.07, 1.29). For SO₂ and PM₁₀, elevated health risks were observed at non-cumulative lags 10, 11, and 12. In the case of SO₂, the cumulative relative risks of CHs were statistically significant at lags 11 through 14, with the highest risk occurring at lag 14 (RR = 1.07). In contrast, cumulative lags for PM₁₀ did not reach statistical significance. Carbon monoxide (CO) showed the highest non-cumulative relative risk at lag 13, indicating a delayed but notable impact on hospitalizations (Figure 1).

Ozone, however, exhibited a distinct exposure-risk pattern compared to other pollutants (Figure 1). A 10 ppb increase in O_3_ concentration was associated with a significantly increased risk of hospitalization from lag 0 to non-cumulative lag 5, after which the risk declined. Importantly, the cumulative relative risks of O₃ exposure remained statistically significant across all lag days (1–14), with the highest observed risk at lag 8 (RR = 1.48).

### 3.4 Gender groups

**Figure 2:**
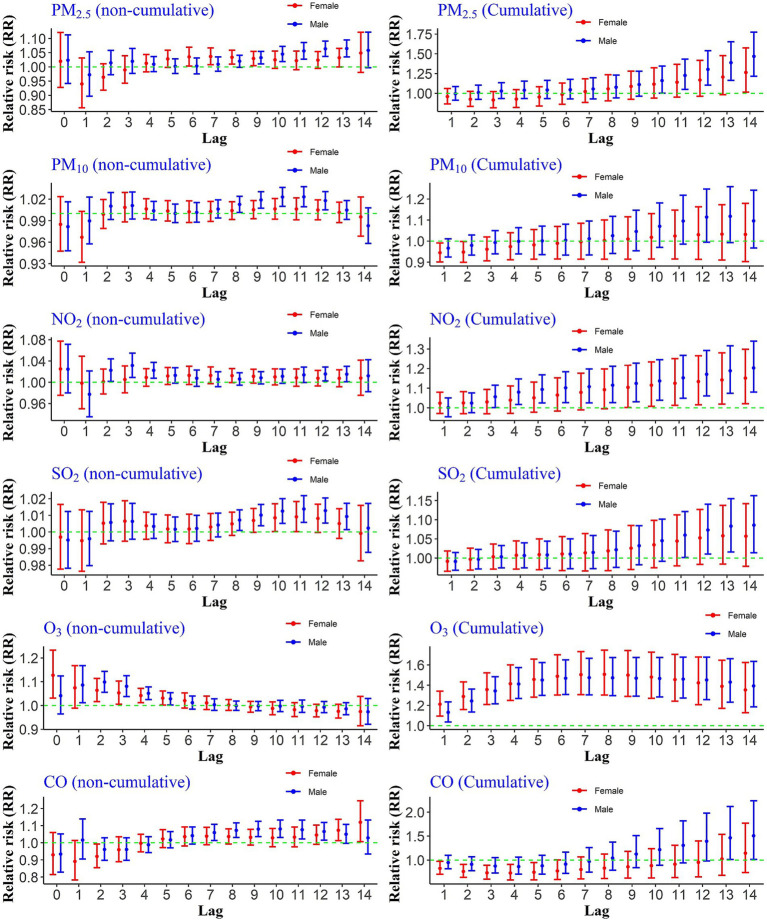
Cumulative and non-cumulative relative risks of CHs for the gender group due to air pollutants across different lag during 2020–2022.

Exposure to PM₂.₅ showed significant non-cumulative effects on hospitalization risks for males at lags 9–13, while significant associations for females were observed at lags 6–9 and again at lag 13. In terms of cumulative exposure, PM₂.₅ was significantly associated with increased hospitalization risk from lags 10–14 among males, and at lag 14 among females (Figure 2).

Exposure to a 10 ppb increase in the concentration of NO₂ significantly increased the relative risks of hospitalization due to COVID-19 for the male group during cumulative lags 4–14. For the female group, significant risks were observed from lags 9–14. The non-cumulative relative risk results for this pollutant indicated a significant risk increase at lags 12, 13, and 14 for the male group. However, the non-cumulative lags were not significant for the female group.

The results for the increase in PM₁₀ concentrations compared to the reference value showed that for the male group, the risk increase was significant at lags 8 to 12, whereas it was not statistically significant for the female group. Additionally, the relative risks of hospitalization due to PM₁₀ exposure during cumulative lags were not significant for either gender. The non-cumulative relative risks of hospitalization due to a 1 ppm increase in CO were significant for males at lags 13 and 14, while no statistically significant increase was observed during either cumulative or non-cumulative lags for the female group.

The effects of O₃ differed from the other pollutants. The non-cumulative relative risk increase occurred immediately for both groups and continued up to lag 5. Moreover, the increase in risk was significant during all cumulative lags (Figure 2).

### 3.5 Age groups

**Figure 3:**
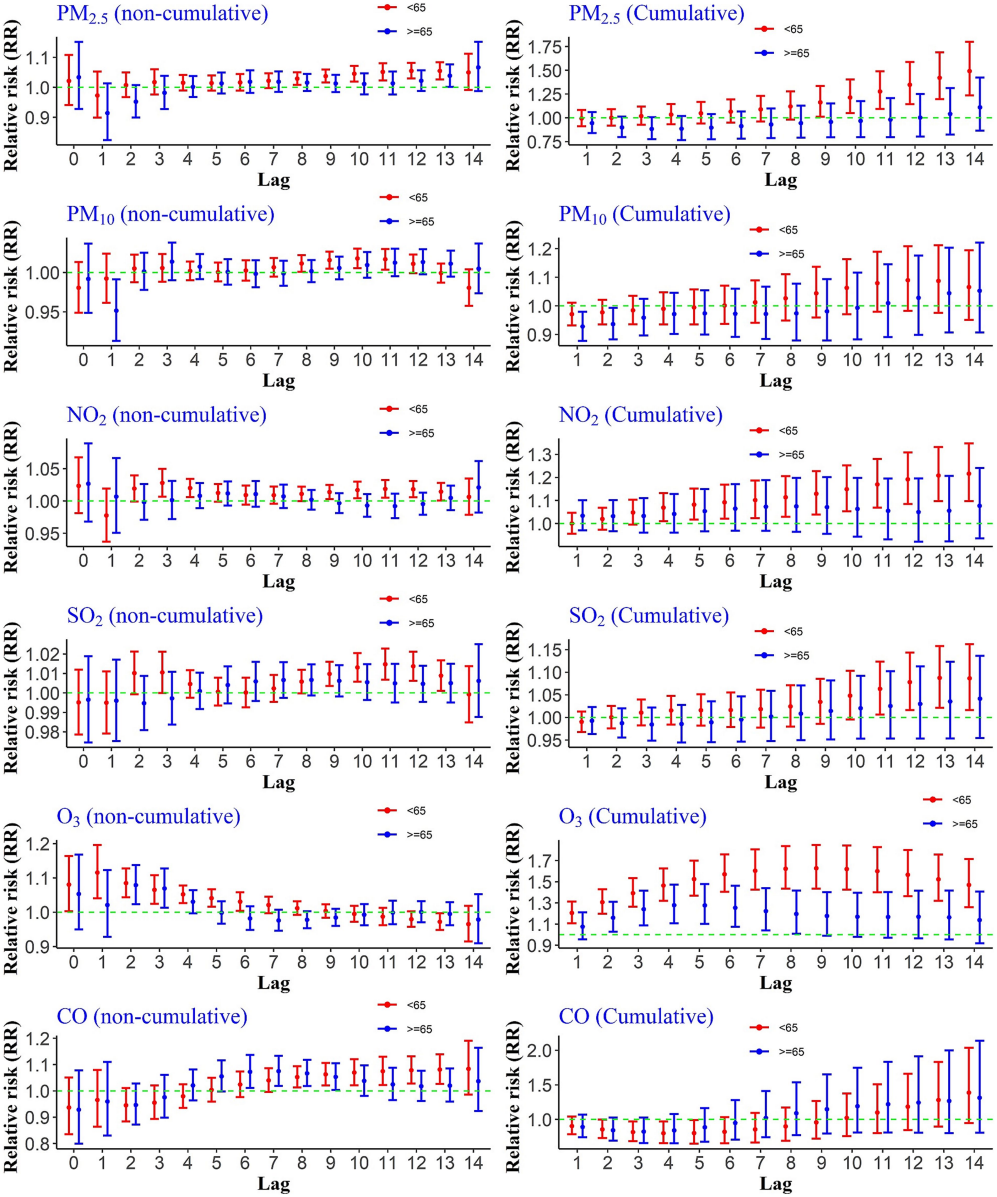
Cumulative and non-cumulative relative risks of CHs for the age group due to air pollutants across different lag during 2020–2022.

To evaluate age-specific differences in the association between air pollution and CHs, we assessed both cumulative and non-cumulative relative risks across age groups (<65 and ≥65 years). As shown in Figure 3, PM₂.₅ exposure was significantly associated with increased non-cumulative hospitalization risk in individuals under 65 at lags 8 through 13, while among those aged 65 and older, a significant effect was observed only at lag 13. For cumulative exposures, significant associations were found at lags 9–14 for the younger age group, whereas no significant effects were observed for the older group. For NO₂ exposure, non-cumulative lags 3, 4, and 9 to 13, as well as cumulative lags 4–14, showed significant associations with hospitalization in individuals under 65. Similarly, PM₁₀ exposure was linked to an increased risk at non-cumulative lags 8 to 11 in the same age group, although no significant cumulative effects were found. SO₂ exposure also demonstrated significant associations with CHs in those under 65 at non-cumulative lags 9–12 and cumulative lags 11–14. In the case of CO exposure, a significant increase in hospitalization risk was found in the younger age group at non-cumulative lags 8–13, while for individuals aged 65 and older, significant associations were observed at lags 6–9. In the case of O_3_, the situation was different. Instantaneously, non-cumulative lag zero (day of occurrence) to lag 6 for people younger than 65 and lag 2 and 3 for people over 65 were significant. Additionally, the risk of hospitalization was statistically significant during all cumulative lags for people under 65 and lags 2–8 for people over 65 years old.

### 3.6 Cumulative lag of 14 days

Cumulative RRs of CHs due to ambient air pollutants over 14 days are presented in Table 2, categorized by the total number of CHs, age, and gender groups. Exposure to these pollutants over the specified 14-day lag period was associated with an increased relative risk of hospitalization. Among the pollutants, the cumulative relative risks for total CHs were highest with exposure to PM₂.₅ and O₃. However, these risks varied across sex and age groups.

**Table 2:** Cumulative relative risks of CHs for the total number and age-gender groups exposed to pollutants over 14 days as lag during 2020–2022.

Air pollutants	Hospitalization				
	Overall	Male	Female	<65	≥65
PM_2.5_	**1.36 (1.15, 1.62) SE = 0.086**	**1.46 (1.21, 1.77) SE = 0.096**	**1.26 (1.01, 1.57) SE = 0.112**	**1.49 (1.23, 1.79) SE = 0.095**	1.10 (0.86, 1.42) SE = 0.126
NO_2_	**1.17 (1.07, 1.29) SE = 0.048**	**1.20 (1.08,1.33) SE = 0.054**	**1.15 (1.02, 1.29) SE = 0.061**	**1.21 (1.09, 1.34) SE = 0.052**	1.07 (0.93, 1.24) SE = 0.071
SO_2_	**1.07 (1.00,1.14) SE = 0.031**	**1.08 (1.01, 1.16) SE = 0.034**	1.05 (0.97, 1.14) SE = 0.039	**1.08 (1.01, 1.16) SE = 0.034**	1.04 (0.95, 1.13) SE = 0.044
PM_10_	1.06 (0.96, 1.18) SE = 0.053	1.09 (0.96, 1.24) SE = 0.063	1.03 (0.90, 1.17) SE = 0.068	1.06 (0.95, 1.19) SE = 0.058	1.05 (0.90, 1.22) SE = 0.075
O_3_	**1.37 (1.19, 1.58) SE = 0.071**	**1.39 (1.18, 1.63) SE = 0.081**	**1.35 (1.12, 1.62) SE = 0.092**	**1.46 (1.25, 1.71) SE = 0.078**	1.13 (0.91, 1.40) SE = 0.108
CO	1.33 (0.93, 1.88) SE = 0.177	**1.50 (1.01, 2.23) SE = 0.201**	1.14 (0.74, 1.77) SE = 0.222	1.38 (0.94, 2.03) SE = 0.194	1.31 (0.80, 2.13) SE = 0.248

The bold values are significant based on 95% confidence interval.

An increase of 10 μg/m^3^ in PM₂.₅ concentration relative to the reference value was associated with a significant increase in RR for males (RR = 1.46; 95% CI: 1.21, 1.77), compared to females (RR = 1.26; 95% CI: 1.01, 1.57). Additionally, the effect of PM₂.₅ was observed in individuals under 65 years of age, but it was not statistically significant for those aged 65 and above. Interestingly, a one-unit increase in CO concentration (1 ppm) had a significant effect on the male group (RR = 1.50; 95% CI: 1.01, 2.23), while no significant associations were found for the female group in relation to CO or SO₂ exposures. Moreover, no significant effects were detected for total CHs with a 10-unit increase in PM₁₀ concentration.

### 3.7 Attributable fraction/number

**Figure 4:**
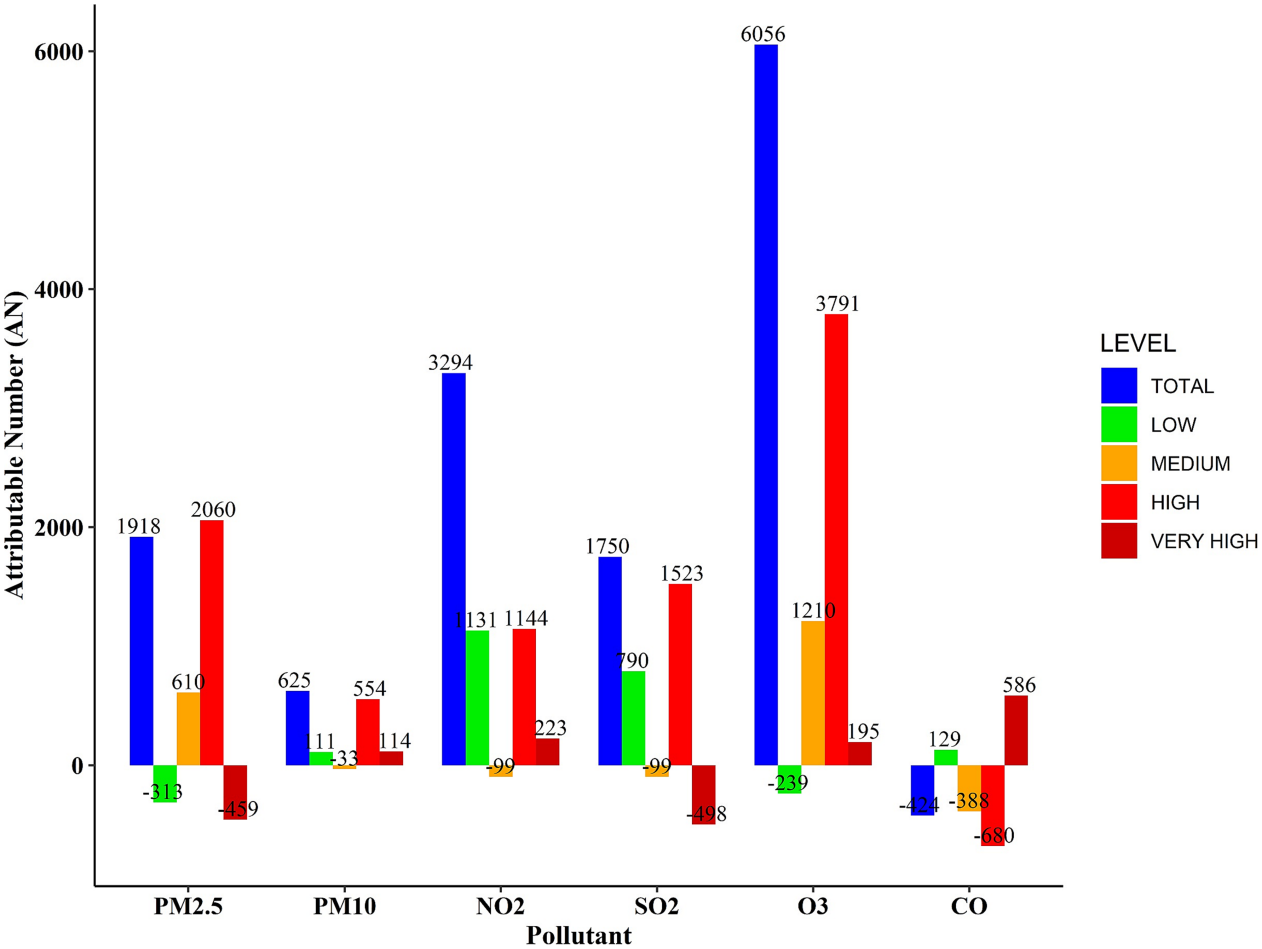
Number of CHs attributed to air pollutants during 2020–2022.

Figure 4 illustrates the number of CHs attributed to air pollutants. The bar chart demonstrates that the highest number of total hospitalizations is attributed to O₃ and NO₂ pollutants, which are responsible for approximately 6,000 and 3,300 hospitalizations, respectively. Analysis across the four concentration ranges reveals that the highest number of CHs is associated with exposures in the high concentration ranges for all pollutants (depicted by the red bars in Figure 4). Very high concentration ranges for PM₂.₅ and O₃ pollutants were not found to be significant, suggesting that the maximum number of hospitalizations cannot be attributed to these extremely high concentration levels. As expected, lower concentration levels of pollutants were linked to fewer hospitalizations (indicated by the green bars in Figure 4).

The AF analysis for air pollutants, along with their corresponding 95% confidence intervals, is presented in Table 3. The AF results were calculated across distinct concentration ranges: total, low, medium, high, and very high. The findings show that significant proportions of hospitalized patients are attributable to PM₂.₅, NO₂, and O₃, with overall concentration ranges accounting for 8.8, 15.2, and 27.9%, respectively.

**Table 3:** The percentage of CHs attributed to air pollutants during 2020–2022.

Pollutants	Overall	Low	Medium	High	Very high
PM_2.5_	**8.8 (0.3, 14.3) SE = 4.33**	8.8 (−2.8, −0.2) SE = 5.91	**2.8 (1.3, 4.1) SE = 0.76**	**9.5 (4.4, 13.9) SE = 2.60**	−1.2 (−4.2, −0.5) SE=1.07
NO_2_	15.2 (8, 22.1) SE=3.67	**5.2 (2.3, 7.6) SE = 1.47**	**4.3 (0.5, 8.0) SE = 1.93**	**5.3 (2.8, 8.6) SE = 1.27**	1.0 (−3, -1.2) SE = 1.12
SO_2_	8.1 (−1.1, 15.9) SE = 4.69	**3.6 (0.4, 6.6) SE = 1.63**	−0.5 (−2.2, −1.1) SE = 0.86	**7.0 (1.1, 12.5) SE = 3.01**	−2.3 (−5.2, 0.2) SE = 1.48
PM_10_	2.9 (−2.1, 6.9) SE = 2.55	0.4 (−1.6, 2.4) SE = 1.02	−0.3 (−2.0, 1.3) SE = 0.86	2.3 (−1.0, 5.8) SE = 1.68	0.5 (−1.5, 2.2) SE = 1.02
O_3_	**27.9 (13.9, 38.8) SE = 7.14**	−1.1 (−2.3, 0.0) SE = 0.61	**5.6 (2.5, 8.1) SE = 1.58**	**17.5 (4.5, 26.8) SE = 6.63**	**7.1 (4.9, 8.8) SE = 1.12**
CO	−1.6 (−13.5, 8.1) SE = 6.07	0.6 (−0.9, 1.7) SE = 0.76	−1.8 (−4.7, 1.1) SE = 1.47	−3.1 (−13.2, 5.4) SE = 5.15	**2.7 (1.1, 4.0) SE = 0.81**

The bold values are significant based on 95% confidence interval.

### 3.8 Sensitivity analysis

Supplementary Table 1 delineates the outcomes of the sensitivity analysis conducted over a 21-day lag period. Overall, the estimated cumulative RRs of air pollutants over this extended lag period were largely consistent with the results obtained for the 14-day lag (Table 2), indicating the robustness of the findings. However, some pollutants exhibited higher effect estimates when the exposure window was extended. For example, the cumulative RR of O₃ for the overall population increased from 1.37 (95% CI: 1.19–1.58) at 14 days to 1.54 (95% CI: 1.31–1.82) at 21 days, suggesting a stronger delayed effect. Similarly, for CO, the RR rose from 1.33 (95% CI: 0.93–1.88) to 1.53 (95% CI: 1.06–2.23), with the association becoming statistically significant only at the 21-day lag. Notably, this increase in risk was more prominent in males and individuals under 65 years of age. These findings underscore the importance of considering longer lag periods in examining the health impacts of air pollution exposure, particularly for pollutants with potential delayed effects.

## 4 Discussion

This study focused on the short-term association between air pollutants and hospitalization due to COVID-19 in Iran. The results showed that air pollutants increase the risk of hospitalization after a 14-day lag.

Evidence from various countries confirms that these pollutants play a role in COVID-19 hospitalizations and deaths. For example, a US-based study found that exposure to PM_2.5_ 7 days before a positive COVID-19 result was associated with a relative risk of hospitalization (RR = 1.18; 95% CI: 1.17, 1.19) (15). Meanwhile, another study showed that a one-unit increase in PM_2.5_ concentration was correlated with an 8% increase in COVID-19 mortality (95% CI: 2–15%) (35). Moreover, research in different European countries has shown that NO_2_ exposure can increase COVID-19 deaths (36). Similarly, a multi-country study across OECD nations found that long-term exposure to elevated PM₂.₅ levels was significantly associated with increased COVID-19 morbidity and mortality at multiple time points during the early stages of the pandemic, further supporting the role of particulate matter as a critical risk factor (18). Other evidence also indicates that the negative impacts of PM_2.5_ and NO_2_ pollutants on COVID-19 hospitalization are consistent with our study’s results (37, 38). In the study area, geographical location and traffic volume are key factors contributing to the continuously high levels of air pollutants throughout the year. The temperature inversion phenomenon is an additional factor that exacerbates pollutant accumulation. Therefore, these pollutants enter people’s bodies daily, worsening respiratory diseases.

Our study, while unable to establish a direct association between PM_10_ and the cumulative relative risks of hospitalization due to COVID-19, is supported by findings from several other studies that reported similar non-significant associations. For example, Jiang et al. examined the effect of ambient air pollutants and meteorological variables on COVID-19 incidence and found that PM_2.5_ and humidity are significantly associated with an increased risk of COVID-19, while PM_10_ and temperature appear to be associated with a decreased risk (39). Similarly, a study reported that PM_10_ did not show a meaningful correlation with COVID-19 hospitalization or mortality, in contrast to other pollutants like NO₂ and PM_2.5_ (40). These results collectively reinforce the notion that PM_10_ may not have a uniformly strong or direct association with COVID-19-related hospitalization or disease severity.

Our study found a positive association between SO₂ exposure and the number of hospitalizations among men and individuals under the age of 65. However, no significant relationship was observed for women or those aged 65 and older. This aligns with some existing evidence, though findings across studies remain inconsistent. For instance, a study involving 33 European countries suggested that SO₂ may negatively impact COVID-19 related morbidity and mortality (41). In contrast, research by Ying reported a negative association between SO₂ and COVID-19 outcomes (42), while another study found no significant relationship between SO₂ exposure and COVID-19 hospitalizations (43).

The AF results for SO₂ indicate that statistically significant associations were observed at low and high concentration levels of this pollutant, while no significant association was found at the medium concentration level. This discrepancy may be attributed to the nonlinear exposure-response relationship captured by the distributed lag nonlinear model (DLNM). In such models, different concentration categories can have varying effects on health outcomes, and not all levels are necessarily associated with statistically significant results.

Among the pollutants examined in this study, O₃ demonstrated the most pronounced impact on COVID-19 hospitalizations. Across the entire concentration range, the attributable fraction of O₃ was approximately 27%, corresponding to over 6,000 hospital admissions. This finding is consistent with results from a large cohort study conducted in Ontario, Canada, which found that long-term exposure to ambient O₃ was significantly associated with higher odds of COVID-19-related hospitalization, ICU admission, and mortality (16, 44). These results highlight ozone as a critical environmental risk factor influencing the progression and severity of COVID-19.

In contrast, our study found that CO had a positive and significant effect only among hospitalized male patients. As a well-known traffic-related air pollutant, CO exposure is often higher among men due to occupational factors that require more frequent presence in outdoor environments. In Iran, this lifestyle pattern results in greater cumulative exposure to air pollution for men compared to women. Interestingly, the number of hospitalizations attributable to carbon monoxide (CO) was positive at both very low and very high concentration levels, while it was negative at medium and high levels, leading to an overall negative attributable number (AN) across the total exposure range. This pattern may suggest a non-linear or potentially U-shaped exposure–response relationship, where both ends of the exposure spectrum are associated with increased health risks, whereas intermediate concentrations may be associated with null or even protective effects. Similar non-monotonic patterns have been previously reported in epidemiological studies investigating air pollution and health outcomes (45).

The observed peak in relative risk at a 14-day cumulative lag in our DLNM analysis likely reflects the delayed and cumulative physiological effects of air pollution on COVID-19 outcomes. Air pollutants such as PM₂.₅ can induce inflammatory responses and compromise immune function effects that may not manifest immediately but accumulate over time, potentially exacerbating the severity of COVID-19. This delayed impact is consistent with findings from a nationwide study in the Netherlands, which reported stronger associations between air pollution exposure and COVID-19 hospital admissions over a 14-day lag compared to a 7-day lag. Specifically, the relative risks for PM₂.₅ and PM₁₀ increased from 1.21 and 1.25 at lag 0–7 to 1.31 and 1.38 at lag 0–14, respectively, indicating a cumulative effect of prolonged short-term exposure (46). Additionally, the typical incubation period of COVID-19, ranging from 2 to 14 days, suggests that exposure to air pollution may influence disease progression and severity after a temporal delay rather than at the point of infection. Moreover, the results of our 21-day lag sensitivity analysis further supported this observation, showing that certain pollutants, such as carbon monoxide (CO), may exert stronger cumulative effects over extended lag periods. Specifically, we found that applying a 21-day lag resulted in a significantly higher relative risk of COVID-19-related hospitalization across all age and gender groups. This pattern suggests that delayed physiological responses such as systemic inflammation, oxidative stress, or immune suppression may require longer exposure windows to manifest their full impact. Similar trends have been reported in other studies; for instance, a multicity analysis conducted in 120 Chinese cities demonstrated that the risk of COVID-19 infection increased progressively over a 21-day exposure window compared to a 7-day period (43). These findings underscore the importance of including extended lag periods in air pollution–COVID-19 models to better capture the delayed and cumulative nature of health effects.

This study identified differential effects of atmospheric pollutants across gender and age groups. The risk estimates for the six pollutants examined were notably higher among males and individuals under the age of 65, compared to females and those aged 65 and above. Previous research has similarly highlighted varying susceptibility to air pollution among COVID-19 patients based on age and gender, although some studies have found these gender-based differences to be statistically non-significant (47). Furthermore, other studies have demonstrated that the relative risk of adverse COVID-19 outcomes linked to air pollution also differs across ethnic and racial groups (48). Additionally, pollutants at moderate and high concentrations were associated with significantly increased risks of COVID-19 hospitalization compared to those at low and extremely high concentrations. The diminished impact observed at very high concentrations may be attributed to their rarity. The infrequent occurrence of both very low and very high pollutant levels likely reduced the statistical power, resulting in nonsignificant findings, as illustrated in Supplementary Figure 2.

In our study, several factors may explain the observed differences in relative risk across sex and age groups: (1) In Iran, men are more likely to spend time outdoors due to occupational responsibilities, whereas a substantial proportion of Iranian women, who are predominantly homemakers, have less exposure to ambient air pollution. This disparity in cumulative exposure likely contributes to the observed differences in risk. (2) Retired adults over the age of 65 tend to spend most of their time indoors, which further limits their exposure to outdoor air pollutants. (3) Women have generally shown higher adherence to public health guidelines compared to men, which may have led to lower COVID-19 infection rates and, consequently, reduced hospitalization rates among women.

Although the present study was conducted in Iran, where strict and prolonged lockdowns were not widely implemented, the findings from countries with more stringent confinement measures offer valuable insights. For example, a study conducted in Spain reported that the short-term impact of air pollutants on COVID-19 related outcomes such as hospital admission and mortality was more pronounced during the lockdown period compared to the post-lockdown phase (49). This was likely due to altered human activities, including reduced industrial operations and transportation, as well as increased indoor heating use. In contrast, Iran’s relatively continuous urban activity may have led to more stable pollution levels, making it challenging to identify distinct periods of exposure. Nevertheless, the Spanish findings support the notion that short-term fluctuations in pollutant levels even during periods of reduced human mobility can significantly affect COVID-19 severity. This reinforces the relevance of closely monitoring air quality, regardless of the presence or absence of lockdown policies.

Overall, our study found that PM_2.5,_ NO₂, and O₃ were the most influential pollutants associated with increased risk of COVID-19 hospitalizations. These findings are consistent with international studies and can be explained by several proposed biological mechanisms. Exposure to PM_2.5_ has been linked to systemic inflammation, oxidative stress, and immune dysfunction, all of which can weaken respiratory defenses and impair the body’s response to viral infections. NO₂ contributes to airway inflammation and epithelial damage, creating a more favorable environment for viral replication. O₃, as a strong oxidant, causes oxidative injury to lung tissue, thereby exacerbating respiratory illness. Furthermore, recent evidence suggests that air pollution may upregulate the expression of ACE2, the receptor used by SARS-CoV-2 to enter human cells (18). Altogether, these mechanisms help explain the significant role of these pollutants in worsening COVID-19 outcomes.

### 4.1 Limitations

Like any scientific research, this study has several limitations. First, the data were obtained from a single hospital in Tehran, which may limit the generalizability of the findings to other regions with different demographic characteristics, environmental conditions, or healthcare infrastructure. Second, due to the lack of residential address data, it was not possible to exclude non-residents who may have traveled from neighboring cities for hospitalization. This could have introduced bias by including individuals with different levels of exposure. Third, while the study focused on the short-term association between ambient air pollution and COVID-19 hospitalization, it did not examine other important outcomes such as infection or mortality. Exploring these outcomes in future studies would provide a more comprehensive understanding of the health burden associated with air pollution during the COVID-19 pandemic.

Additionally, due to data limitations, potential confounding factors such as population density, socioeconomic status, underlying health conditions, and access to healthcare services were not accounted for, although they may influence both exposure and outcomes. Moreover, the study assessed only ambient air pollution, and did not consider indoor exposure, which could affect the accuracy of exposure estimates especially for older adults who spend most of their time indoors. Therefore, the possibility of exposure misclassification should be acknowledged. Future studies are encouraged to incorporate individual-level exposure data and adjust for a broader range of confounding variables to enhance the accuracy and generalizability of results.

## 5 Conclusion

In conclusion, our study demonstrates a significant association between exposure to certain ambient air pollutants specifically PM₂.₅, NO₂, and O₃ and the risk of hospitalization due to COVID-19 in Tehran. These findings were especially pronounced among males and individuals under the age of 65, likely due to increased outdoor activity and, consequently, greater pollutant exposure. In contrast, no significant association was observed with PM₁₀ or SO₂.

This evidence highlights the disproportionate health risks faced by specific population subgroups and underscores the urgent need for targeted public health interventions. Effective air quality management policies such as reducing emissions from traffic and industrial sources, promoting cleaner transportation, enforcing stricter emission standards, and increasing public awareness are critical to mitigating the health burden of air pollution. Furthermore, incorporating environmental health strategies into pandemic preparedness plans is essential for protecting vulnerable populations in future health crises.

## Data Availability

The raw data supporting the conclusions of this article will be made available by the authors, without undue reservation.

## Footnotes

^1^ http://aqms.doe.ir/

^2^ https://airnow.tehran.ir/
