# Supplementary figures and images for: Assessing the risks of short-term exposure to ambient air pollutants on COVID-19 hospitalizations in Tehran, Iran: a time-stratified case-crossover approach
Source: Front Public Health. 2025 Jun 3;13:1514721. doi: 10.3389/fpubh.2025.1514721 (PMC12170561; doi:10.3389/fpubh.2025.1514721)

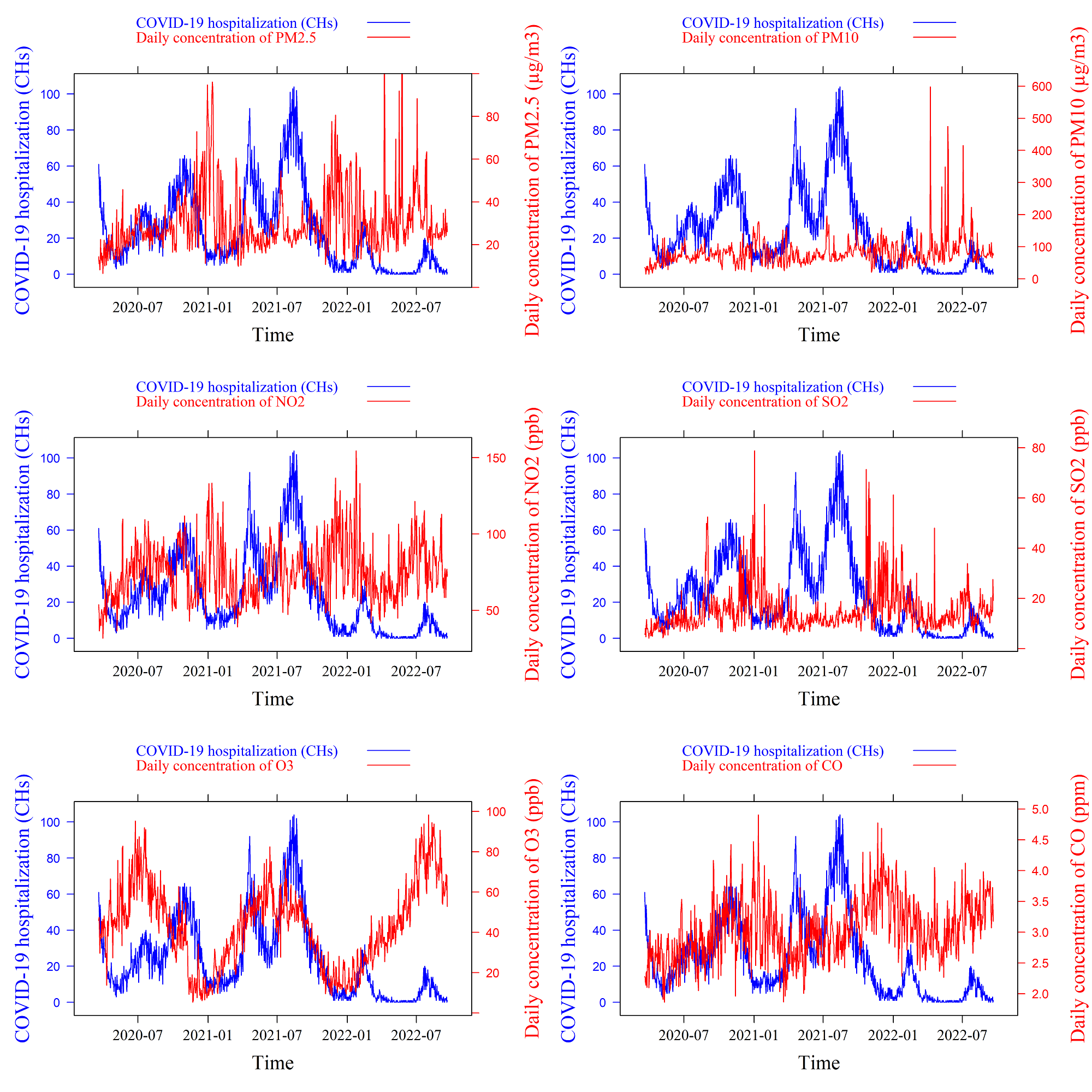

Supplement: Supplementary file 1 [file Image_1.TIF]

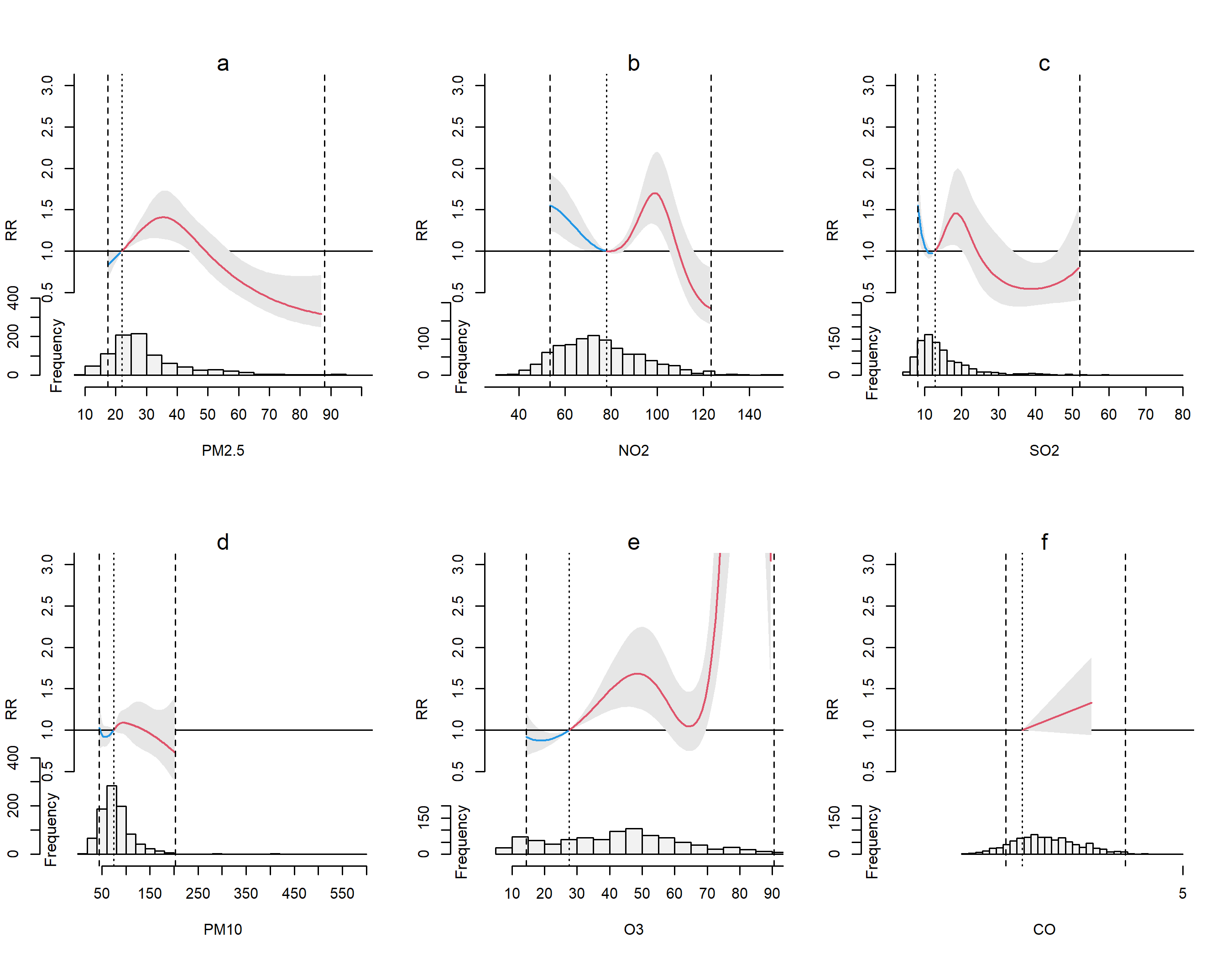

Supplement: Supplementary file 2 [file Image_2.PNG]
