# Supplementary material for: Assessing the risks of short-term exposure to ambient air pollutants on COVID-19 hospitalizations in Tehran, Iran: a time-stratified case-crossover approach
Source: Front Public Health. 2025 Jun 3;13:1514721. doi: 10.3389/fpubh.2025.1514721 (PMC12170561; doi:10.3389/fpubh.2025.1514721)
Supplement: Supplementary file 3 [file Table_1.DOCX]

**Supplementary Table 1.** Cumulative relative risks of hospitalization of patients exposed to air pollutants during 21 days as a time lag during the study period (2020-2022).

| Air pollutants | Hospitalization | | | | |
| --- | --- | --- | --- | --- | --- |
|  | Overall | Male | Female | <65 | >=65 |
| PM2.5 | **1.45(1.14, 1.85)**  **SE=0.124** | **1.49(1.13, 1.95)**  **SE=0.140** | **1.43(1.05, 1.95)**  **SE=0.158** | **1.54(1.18, 2.00)**  **SE=0.135** | 1.32(0.91, 1.90)  SE=0.189 |
| NO2 | **1.21(1.07, 1.37)**  **SE=0.062** | **1.22 (1.06,1.41)**  **SE=0.071** | **1.21(1.03, 1.41)**  **SE=0.082** | **1.27(1.11, 1.45)**  **SE=0.068** | 1.08(0.89, 1.31)  SE=0.098 |
| SO2 | **1.08 (0.99,1.17)**  **SE=0.044** | **1.08(0.99, 1.18)**  **SE=0.044** | 1.08(0.97, 1.19)  SE=0.054 | **1.09(0.99, 1.19)**  **SE=0.049** | 1.06(0.94, 1.19)  SE=0.061 |
| PM10 | 0.96(0.85, 1.09)  SE=0.062 | 0.99(0.87, 1.13)  SE=0.065 | 0.93(0.79, 1.08)  SE=0.083 | 1.00(0.87, 1.14)  SE=0.071 | 1.92(0.77, 1.10)  SE=0.09 |
| O3 | **1.56(1.32, 1.84)**  **SE=0.085** | **1.57(1.29, 1.90)**  **SE=0.1** | **1.55(1.25, 1.92)**  **SE=0.1** | **1.66(1.38, 1.99)**  **SE=0.094** | **1.28(1.00, 1.65)**  **SE=0.125** |
| CO | **2.15(1.31, 3.51)**  **SE=0.252** | **2.49(1.41, 4.40)**  **SE=0.290** | 1.78(0.95, 3.34)  SE=0.320 | **2.28(1.32, 3.95)**  **SE=0.278** | **2.11(1.05, 4.24)**  **SE=0.356** |
